# Inducing Enantioselectivity in Radiative Charge-Transfer Rates through Chiral Quantum Electrodynamic Fluctuations

**DOI:** 10.1021/acs.jpclett.6c02328

**Published:** 2026-10-05

**Authors:** Yu-Chen Wei, Liang-Yan Hsu

**Affiliations:** † Department of Chemistry, 34881 National Tsing Hua University, Hsinchu 300044, Taiwan; ‡ 71556 Institute of Atomic and Molecular Sciences, Academia Sinica, Taipei 106, Taiwan; ¶ Department of Chemistry, National Taiwan University, Taipei 106, Taiwan; § National Center for Theoretical Sciences, Taipei 106, Taiwan

## Abstract

Chiral discrimination has long been an important yet challenging topic in chemistry and physics. In this work, we study how chiral light–matter interaction under weak coupling regime enhances enantioselectivity of chiral charge transfer (CT) rates. Based on the framework of macroscopic quantum electrodynamics and Marcus electron transfer theory, we predict that the degree of discrimination (*S*) of a single-molecule asymmetric radiative CT reaction can achieve −2.0 × 10^–2^ through the light–matter interactions between a silicon nanohelix and a helicene-based CT dye. Orientation dependence of *S* is systematically analyzed, showing the averaged *S* < −10^–3^ for an isotropically oriented molecule. We further demonstrate that different molecular positions influence the orientation dependence of *S*. The results demonstrate how chiral vacuum electromagnetic fields influence enantioselectivity of radiative CT rates, opening up a new route to achieve chiral discrimination in molecular CT.

## Graphical abstract

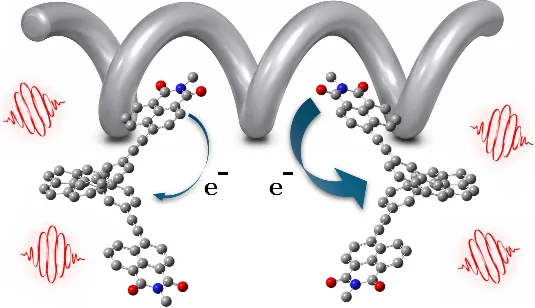

Chiral discrimination plays a crucial role across diverse chemical, physical, and biological processes, most prominently in enantioseparation of pharmaceutical compounds or biomolecules^1,2^ and in phenomena such as chiral-induced spin selectivity^3,4^ and valley transport.^5,6^ Broadly, the driving forces underlying separation strategies can be categorized into two main classes. The first involves static electromagnetic interactions between chiral entities, which form the basis of traditional approaches such as crystallization and chromatographic techniques,^7,8^ where intermolecular forces and stereospecific recognition guide the selective partitioning of enantiomers. The second category relies on electrodynamic interactions, in which external fields (such as electric,^9,10^ magnetic^11,12^ or optical fields^13,14^ ) are applied to actively manipulate chiral systems.

Recently, strong light–matter interactions mediated by quantum electromagnetic fluctuations have been proposed as a promising noninvasive strategy for manipulating enantioselectivity, circumventing the need for externally applied static or dynamic fields.^15–19^ In such systems, the coupling between chiral photons and material excitations gives rise to chiral polaritons, quasiparticles that simultaneously inherit the handedness of the photonic mode and the matter-specific properties of the exciton. This hybrid nature endows chiral polaritons with long-range spatial coherence and handedness, which draws great interest in optoelectronics and quantum technologies.^15,16,20–23^ Notably, the spatial coherence property has been extensively validated in a range of photonic architectures, including Fabry–Perot microcavities,^24,25^ distributed Bragg reflector,^26,27^ metasurfaces,^28,29^ and two-dimensional materials,^30,31^ where ballistic transport and extended propagation lengths have been observed even under ambient conditions. To date, chiral polaritons have been experimentally demonstrated in engineered photonic structures to exhibit chiral lasing^32–34^ and other chiral nonlinear optical effects.^35–39^

Among the above demonstrations, enantioselectivity has been induced through the formation of chiral strong coupling. However, maintaining the strong-coupling regime is challenging, as the environmental dielectric function continuously evolves during chemical reactions.^40–42^ These limitations underscore the difficulty of controlling chemical reactivity via strong light–matter coupling. In this study, we investigate how chiral light–matter interactions in the weak-coupling regime influence the enantioselectivity of chiral charge-transfer (CT) reactions, with particular emphasis on the role of the chiral Purcell effect on radiative CT processes. Note that chiral Purcell effect, enhancements of local chiral photonic density of states, plays a promising role in developments of biomolecular sensing^43,44^ and chiral optoelectronic devices.^45,46^

To appropriately describe quantum electromagnetic fluctuations in chemical systems, we model a molecule coupled to a structured photonic environment within the framework of macroscopic quantum electrodynamics (QED), which has been used to extend several classical chemical theories,^47^ including molecular fluorescence,^48^ energy transfer,^49^ nonadiabatic processes,^50^ superradiance,^51,52^ and electron transfer.^53^ Here we extend our previous macroscopic quantum electrodynamic electron transfer (mQED-ET) theory^53^ and predict chiral CT rates in a donor–acceptor dyad. Specifically, we analyze the degree of discrimination of radiative chiral CT in a helicene-based acceptor–donor–acceptor system interacting with a silicon nanohelix. This work provides mechanistic insight and design principles for achieving quantum-electrodynamic-controlled chiral discrimination in the weak light–matter coupling regime.

## Theoretical Framework

To properly describe the light–matter interaction in a dispersive, absorbing, and asymmetric dielectric environment, we start from the length-gauge Hamiltonian in the framework of macroscopic QED including the contribution of magnetic field.^54–56^

1 $$Ĥ=\underset{\alpha }{\sum }\frac{{\mathrm{p̂}}_{\alpha }^{2}}{2m_{\alpha }}+\frac{1}{2}\int dr{\mathrm{\rho ̂}}_{M}\left(r\right)\cdot {\mathrm{\phi ̂}}_{M}\left(r\right)+\int dr\int_{0}^{\infty }d\omega \hbar \omega {\mathrm{f̂}}^{†}\left(r,\omega \right)\cdot \mathrm{f̂}\left(r,\omega \right)-\mathrm{\mu ̂}\cdot Ê\left(r_{M}\right)-\mathrm{m̂}\cdot \mathrm{B̂}\left(r_{M}\right)$$

*m*_α_ and **p̂**_α_ indicate the mass and the momentum operator of the αth particle, respectively. ρ̂_M_(**r**) represents a charge density operator for the molecule. ϕ̂_M_(**r**) corresponds to molecular Coulomb potential operator. **f̂** ^†^(**r**, ω) (**f̂**(**r**, ω)) is the creation (annihilation) operator for bosonic vector fields (polariton) in macroscopic QED.^54–56^ Here the polariton refers to the hybridization of the photon and the polarization of the media, which does not imply the formation of strong light-molecule coupling.^50^ The term **μ̂**·**Ê**(**r**_M_) and **m̂**·**B̂**(**r**_M_) describe the light–matter interaction in the dipole form, where **μ̂**, **Ê**(**r**_M_), **m̂**, and **B̂**(**r**_M_) represent the electric dipole moment operator, the electric field operator, the magnetic dipole moment operator and the magnetic field operator, respectively. Note that the quadratic terms are dropped due to their negligible effects on coupling strength and radiative ET rates.^53,57^ According to the study of polaritonic Huang–Rhys factor, the dipole self-energy can be transformed, via a polaron-type unitary transformation, into reorganization dipole self-coupling terms that appear only in the off-diagonal matrix elements.^57^ Furthermore, the mQED-ET theory demonstrated that the contribution of this reorganization dipole self-coupling to QED-driven ET rates is negligible.^53^ These results indicate that neglecting the dipole self-energy does not lead to an energy divergence and has no significant influence on the calculated CT rates.

To obtain the effective light–matter coupling, we follow the derivation of the mQED-ET theory.^53^ First, we develop the model Hamiltonian considering electronic, vibrational and polaritonic degrees of freedom [see Supporting Information section S1]. We consider a two-state molecular model in the electronic subspace with a harmonic vibrational bath based on the standard molecular ET theory.^58,59^ After the small-polaron transformation [see Supporting Information section S2], we obtain the effective CT coupling ${\mathrm{V̂}}_{\mathrm{CT}}$ by collecting all the electronic off-diagonal terms together.

2 $${\mathrm{V̂}}_{\mathrm{CT}}\equiv \left(V_{CT}-d_{\mathrm{CT}}\cdot Ê\left(r_{M}\right)-m_{\mathrm{CT}}\cdot \mathrm{B̂}\left(r_{M}\right)\right)\left|\mathrm{DA}\right\rangle \left\langle D^{+}A^{-}\right|{\mathrm{D̂}}_{\mathrm{vib}}+\mathrm{h.c.}$$

where *V*_CT_ is the Coulomb electronic coupling, **d**_CT_ and **m**_CT_ are the transition electric dipole moment and transition magnetic dipole moment of a CT transition, and *D̂*_vib_ is the vibrational displacement operator. Based on ${\mathrm{V̂}}_{\mathrm{CT}}$ in eq 2, we apply the Fermi’s golden rule to evaluate ET rate in the electronic weak-coupling approximation and the light-matter weak-coupling approximation. Next, based on the same approximations in mQED-ET theory including the Condon approximation, the short-time approximation, and the low-vibrational-frequency limit,^53,60^ we derive an explicit expression for the total CT rate *k*_CT_ as *k*_CT_ = *k*_Marcus_ + *k*_QED_. The detailed derivation can be found in Supporting Information section S3. *k*_Marcus_ corresponds to the Marcus nonradiative ET rate.^53,60,61^ *k*_QED_ is the QED-driven radiative ET rate composed of two terms.

3 $$k_{\mathrm{QED}}=k_{\mathrm{el}}+k_{\mathrm{ch}}$$

*k*_el_ corresponds to the radiative CT rate caused by the transition electric dipole moment only, which agrees with the mQED-ET theory.^53^ *k*_ch_ gives rises to the chiral CT response determined by

4 $$k_{\mathrm{ch}}=\frac{2\pi }{\hbar }\int_{0}^{\infty }d\omega J_{\mathrm{ch}}\left(\omega \right)\rho_{\mathrm{vib}}\left(\omega \right)$$

where ρ_vib_(ω) represents the vibrational transitiona density of states (DOS) and *J*_ch_(ω) corresponds to the chiral spectral density expressed as follows.

5 Jch(ω)≡2ℏωμ0π⁡Im[mCT·∇×Im⁡G®®(rM,rM,ω)·dCT*]

Im⁡G®®(rM,rM,ω) is the imaginary part of the local dyadic Green’s function, which is associated with the local photonic DOS (local density of optical states).^62,63^ Note that the expression of *k*_ch_ agrees with the spontaneous emission in the chiral environment (chiral Purcell effect).^64^ In other words, both chiral spontaneous emission and chiral CT are associated with the same chiral spectral density, while they are different from the charge displacements during the electronic displacements. It is obvious that the achiral dielectric environment (∇×Im⁡G®®(rM,rM,ω) = 0) leads to *k*_ch_ = 0, and the theory would be reduced to the mQED-ET theory considering the interaction from **μ̂**·**Ê**(**r**_M_) only.^53^ Note that achiral molecules with zero rotational strength Im­[**m**_CT_·**d**_CT_^*^] = 0 do not guarantee *k*_ch_ = 0. In addition, different handedness of molecules give rise to opposite sign of **d**_CT_, leading to the chiral discrimination in chiral CT processes shown as follows.

6 $$k_{\pm }=k_{\mathrm{el}}\pm k_{\mathrm{ch}}$$

where *k*_±_ represent the chiral CT rates of the two enantiomers. To quantify the degree of chiral discrimination, we define the parameters *S* as^64,65^

7 $$S=\frac{k_{+}-k_{-}}{k_{+}+k_{-}}=\frac{k_{\mathrm{ch}}}{k_{\mathrm{el}}}$$

It is worth noting that the physical meaning of *S* differs fundamentally from that of the dissymmetry factor *g*, which characterizes circularly polarized light. Specifically, *g* quantifies the relative difference between the radiative rates of right- and left-handed circularly polarized light emitted by an enantiomeric system,^66,67^ whereas *S* quantifies the relative difference in the transition rates of the two enantiomers for a system initially prepared as a racemic mixture.

## Numerical Methods

Although eqs 4 and 5 provide the working expression for evaluating *k*_ch_, directly calculating the term

Im[mCT·∇×Im⁡G®®(rM,rM,ω)·dCT*]

in an arbitrary chiral dielectric environment remains challenging.^64^ To address this issue, we develop a numerical framework that integrates quantum chemistry calculations with electrodynamics simulations to obtain *J*_ch_(ω) (Figure 1). First, **d**_CT_, **m**_CT_, and the angle between them, denoted by α, are obtained from quantum chemistry calculations. Next, the chiral dipoles are constructed in the electrodynamics simulation software based on the calculated dipole moments. For a given dielectric environment, the chiral radiative rates of the two enantiomeric dipoles can then be evaluated. Note that these chiral radiative processes do not include vibrational relaxation, although they still contain the information required to determine the chiral spectral density. Using eq 6 together with eqs S41 and S42, the electric and chiral components of the CT spectral density, *J*_el_(ω) and *J*_ch_(ω), can be extracted. By subsequently incorporating ρ_vib_(ω) into the working equations of transition rates (eqs S41 and S42), one can readily evaluate *k*_el_, *k*_ch_, and *S*. This method provides a versatile approach for handling the complex form of *J*_ch_(ω) and can be broadly applied to calculate *S* arising from chiral light–matter interactions in arbitrary dielectric environments.

**Figure 1:**
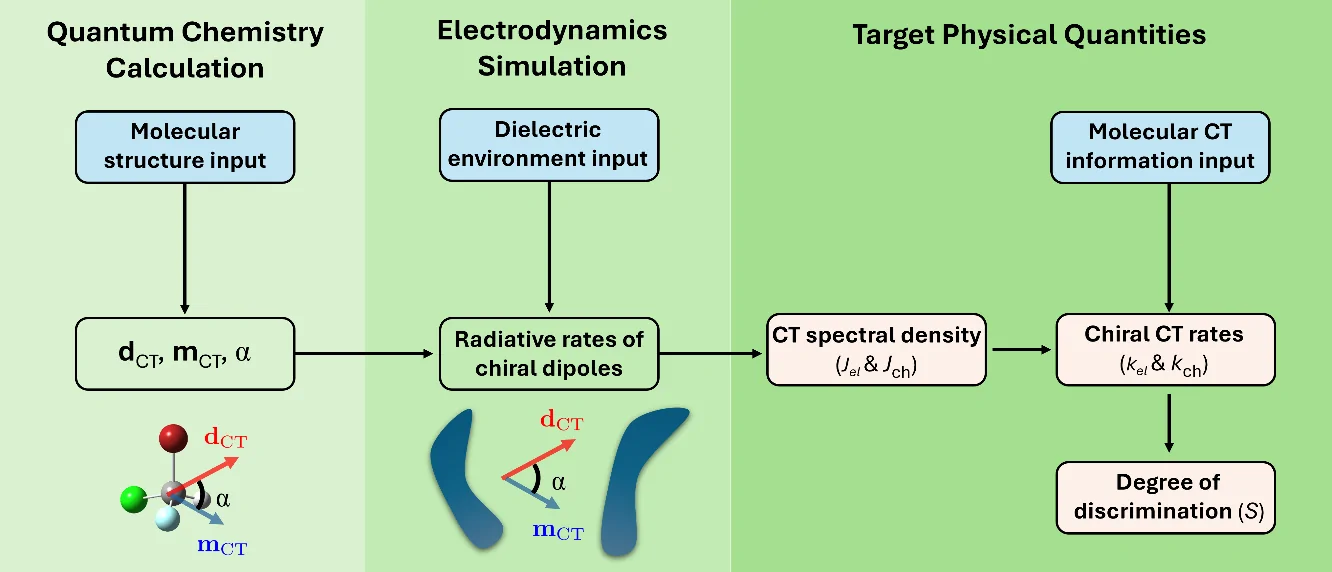
Flowchart of the computation of *J*_ch_(ω), *k*_ch_, and *S*. The blue and orange boxes represent the input and output information, respectively.

## Example Demonstration

To examine the effect of chiral light–matter interaction on enantioselectivity, we consider a helicene-based CT dye composed of a helicene and a naphthalimide as a model system for investigating chiral charge-recombination processes (Figure 2a).^68^ Upon charge recombination, electron transfer occurs from the naphthalimide moiety to the helicene moiety, as revealed by the frontier molecular orbital analysis (Figure 2b). According to the previous study,^68^ |**d**_CT_| = 9.28 D, |**m**_CT_| = 2.76 au, and α = 68.2° are used. In addition, the high photoluminescent quantum yield (PLQY = 45%) and radiative rate (*k* = 4 × 10^8^ s^–1^) in cyclohexane make this molecule a promising candidate for manipulating radiative CT rates through light–matter interactions. Note that significant QED-driven charge-transfer effects require the radiative CT pathway to be sufficiently competitive with the nonradiative pathway,^53,60^ and this study focus on the chiral discrimination of radiative pathways. In addition, the involvement of nonradiative processes would decrease the observed *S*. For the molecular system considered here, with the observed PLQY of 45%, the observable *S* is estimated to be approximately half of the calculated value for the radiative CT process alone. The two enantiomers, (P)-ADA and (M)-ADA, possess identical **m**_CT_ but **d**_CT_ with opposite signs.^69^ In isotropic, parity-symmetric free space, the two enantiomers should have identical total spontaneous-emission rates. Based on our method, a degree of chiral discrimination of *S* = 1.2 × 10^–6^ (*k*_el_ = 3. 0 × 10^8^ s^–1^; *k*_ch_ = 3.9 × 10^2^ s^–1^) is evaluated in free space, which is the residual of the numerical subtraction. In the following sections, we introduce chiral light–matter interactions into the system to investigate their influence on *S*.

**Figure 2:**
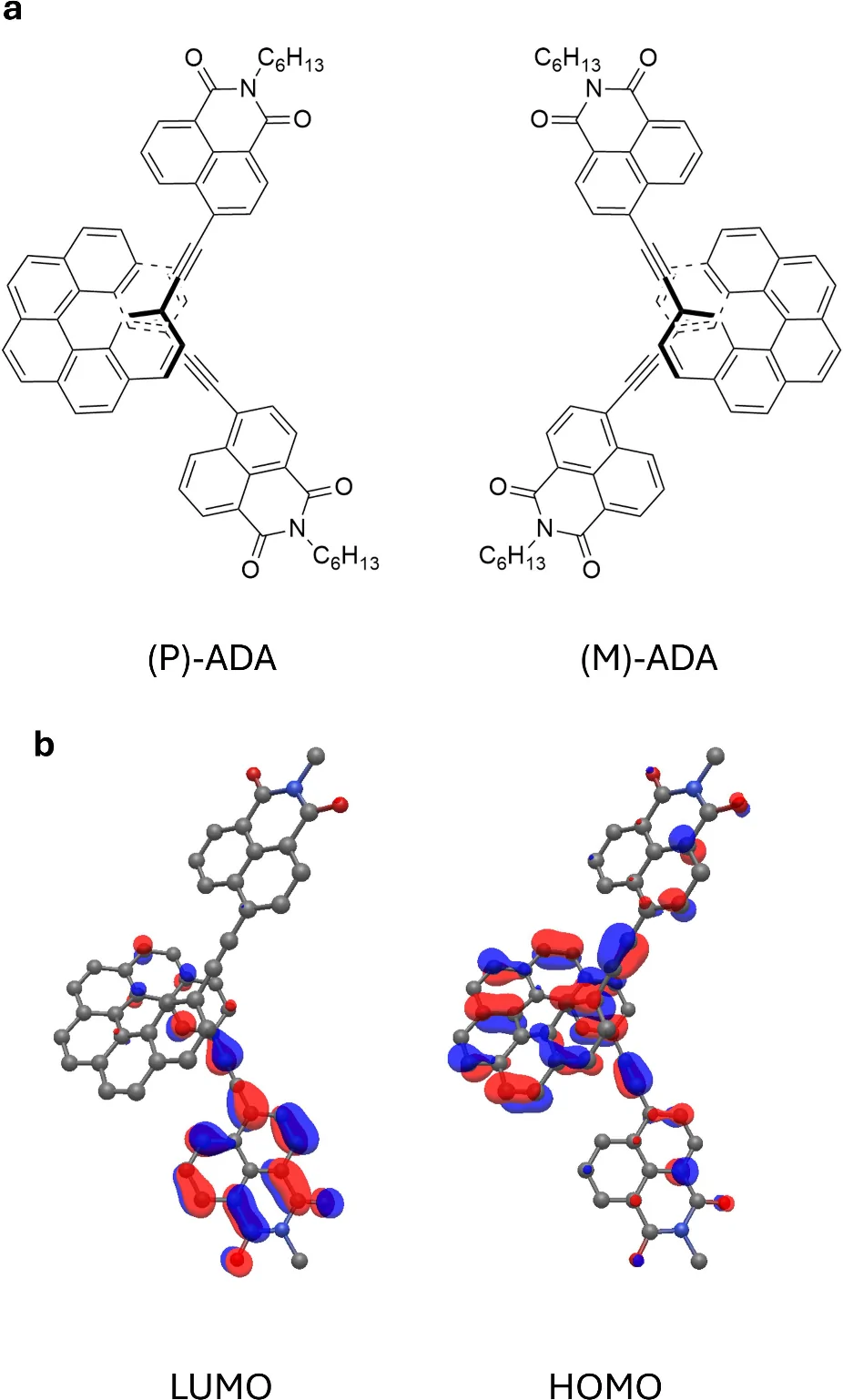
Candidate molecule for enantioselective CT reaction via weak chiral light-matter interaction. (a) Chemical structure of the enantiomers of the helical push–pull dyes (P)-ADA and (M)-ADA. (b) Isosurfaces of frontier molecular orbitals of (P)-ADA. The orbital configuration contributes 95% to the S_1_–S_0_ transition. The molecular geometry in (b) is optimized in the S_1_ charge-transfer state. See Computational Methods for detailed information on the quantum chemical calculation.

The chiral light–matter interaction is engineered via the Si nanohelix whose structure is shown in Figure 3a. Worthy to mention, Si nanohelices with similar geometries have previously been employed to enhance local optical chirality and to demonstrate chiroptical second-harmonic Tyndall scattering effects.^70^ Here we use a Si nanohelix to generate chiral QED fluctuations and induce enantioselectivity in the charge-transfer (CT) processes of (P)-ADA and (M)-ADA. (Figure 3b). Here the discrimination in CT rates between the two enantiomers does not rely on any external fields, as it is induced by the vacuum fluctuations of the chiral polaritonic modes supported by the Si nanohelix. Based on the Si nanohelix and the defined molecular position (center of the nanohelix), the orientation dependence of the degree of discrimination *S* is evaluated (Figure 3c). Among different molecular orientations, the molecular dissipation estimated from the emission peak width (full width half-maximum of ≈0.6 eV) exceeds the coupling strengths estimated from the Purcell factor (≈10^–7^ eV),^48^ confirming that the system is within the weak light–matter coupling regime. The numerical accuracy is demonstrated in Figure S1, which shows that the calculated observable is well converged with respect to the parameters used in our calculations. The corresponding *k*_el_ and *k*_ch_ are shown in Figure S2. To further verify the calculated handedness, the opposite handed helix and the achiral structure (Si thin film with the thickness of 250 nm) are considered. When the molecule is at the center of the helix and the molecular electric dipole is aligned along the *z*-axis, the two handednesses of the helix yield *S* = 6.0 × 10^–3^ and −5.9 × 10^–3^, respectively, consistent with the enantiomeric structures exhibiting comparable magnitudes of chiral discrimination but opposite signs. In addition, *S* = 2.1 × 10^–6^ is obtained when the molecule is positioned 1 μm above the Si thin film with the thickness of 250 nm, which is very close to the residual obtained in free space. Note that the strong near field interaction between a chiral dipole and an achiral medium would induce chirality via symmetry breaking.^71^ To verify the difference between molecular chiral CT and chiral Purcell effect, we analyze the spectral dependence of *J*_ch_/*J*_el_ (Figure S3a). The results show that *J*_ch_/*J*_el_ exhibits a pronounced spectral peak within the wavelength range where the vibrational transition DOS is significant. Notably, the peak value of *J*_ch_/*J*_el_ (−7.8 × 10^–3^) differs from the resulting degree of chiral discrimination, *S* = −5.9 × 10^–3^, after accounting for vibronic dissipation. This discrepancy highlights an important distinction between radiative chiral CT processes in molecules and the chiral Purcell effect, as the former involves spectral averaging weighted by the molecular vibrational transition DOS. The individual spectral dependence of *J*_ch_ and *J*_el_ are further presented in Figure S3b. Their spectral profiles demonstrate that vibronic weighting can substantially modify their respective contributions to the *k*_el_ and *k*_ch_ and, consequently, to *S*. These results clearly highlight the essential role of molecular vibronic dissipation in determining the magnitude of chiral discrimination in CT processes.

**Figure 3:**
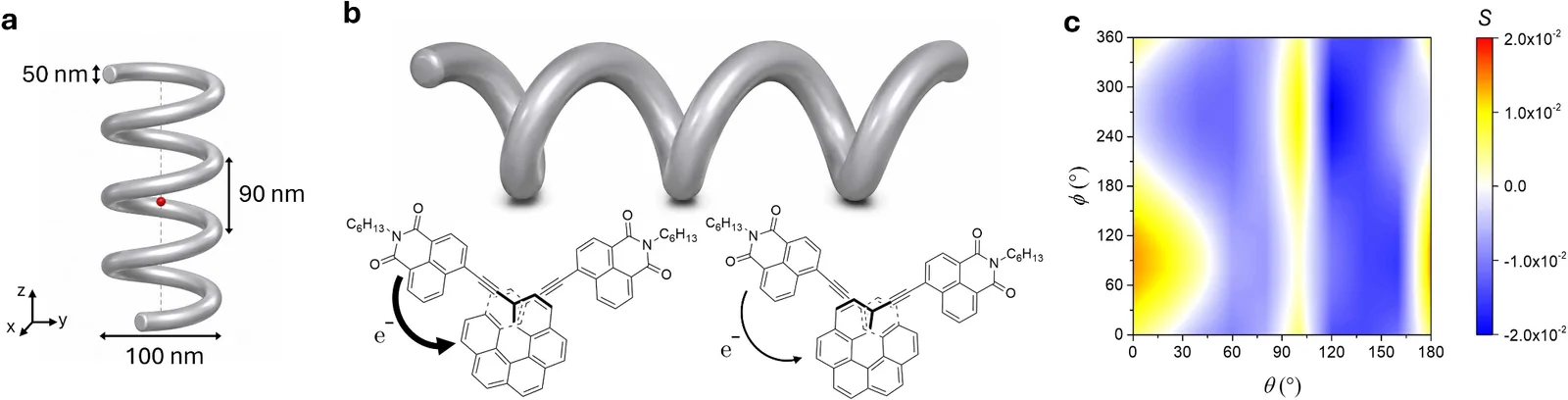
Manipulating enantioselective CT rates via chiral light–matter interaction of a silicon helix. (a) Schematic illustration of the silicon nanohelix. The dimensions of the helix are height 270 nm, pitch 90 nm, wire radius 25 nm, and loop radius 50 nm. The red dot, the center of the helix, indicates the position of the molecule. (b) Schematic illustration of chiral discrimination in charge recombination in (P)-ADA and (M)-ADA. The thickness of the arrows indicates the CT rates. (c) Orientation dependence of *S* for the molecule located in the center of the helix. θ and ϕ are the polar angle and the azimuthal angle of the direction of **d**_CT_.

The minimum value *S* = −2.0 × 10^–2^ is obtained at (θ, ϕ) = (120°, 280°), representing an approximately two-orders-of-magnitude enhancement over the free-space value. Such order of magnitude of *S* is measurable via time-resolved chiral spectroscopy to resolve the chiral discrimination in asymmetric CT kinetics.^72–75^ Regions with pronounced discrimination (*S* < −10^–2^) are mainly distributed within (θ = 120°–150°), corresponding to molecular orientations in which **m**_CT_ is approximately aligned along the *z*-axis. In contrast, the local minimum observed within the region of θ = 30°–90° exhibits a pronounced dependence on ϕ. Notably, averaging over all molecular orientations yields *S* = −(9.2 ± 8.5) × 10^–3^, indicating that the right-handed Si nanohelix preferentially favors *S* < 0 even when the molecules undergo isotropic rotational motion in solution. Here we evaluate the ensemble-averaged *S* via averaging the CT rates over the ensemble and then calculating their ratio. In addition, the deviations here indicate the spread across molecular orientations rather than uncertainty in the calculated mean. Moreover, it is reasonable to neglect the purely magnetic contribution in our system, as its magnitude is approximately 10^–2^–10^–3^ smaller than that of *k*_ch_.

Since molecules diffuse randomly in solution, it is important to examine the spatial dependence of *S*. In Figure 4, four molecular positions are considered: (*x*, *y*, *z*) = (0 nm, 0 nm, 45 nm), (0 nm, 0 nm, 90 nm), (10 nm, 0 nm, 0 nm), and (20 nm, 0 nm, 0 nm). The positions (0 nm, 0 nm, 45 nm) and (0 nm, 0 nm, 90 nm) correspond to vertical displacements of one-half pitch and one full pitch of the helix, respectively. The corresponding *k*_el_ and *k*_ch_ are shown in Figure S4. Note that the molecule is separated from the Si boundary by 27.5 and 17.5 nm when displaced by 10 and 20 nm along the *x*-axis, respectively (Figure S5). The results show that the highly discriminating region at θ = 120°–150° is largely insensitive to vertical displacement, whereas the region at θ = 30°–90° changes substantially. Compared with Figure 3b, the local minimum in the θ = 30°–90° region shifts by approximately 180° in the ϕ-domain for the two displaced positions (Figure 4a,b). In contrast, the robust *S* are observed within θ = 120°–150°. A similar trend is also observed when the molecule is displaced along the *x*-axis. As the in-plane displacement increases, the local minimum around (θ, ϕ) ≈ (60°, 60°) gradually disappears, whereas the shape and position of the minimum within (θ = 120°–150°) remain nearly unchanged (Figure 4c,d).

**Figure 4:**
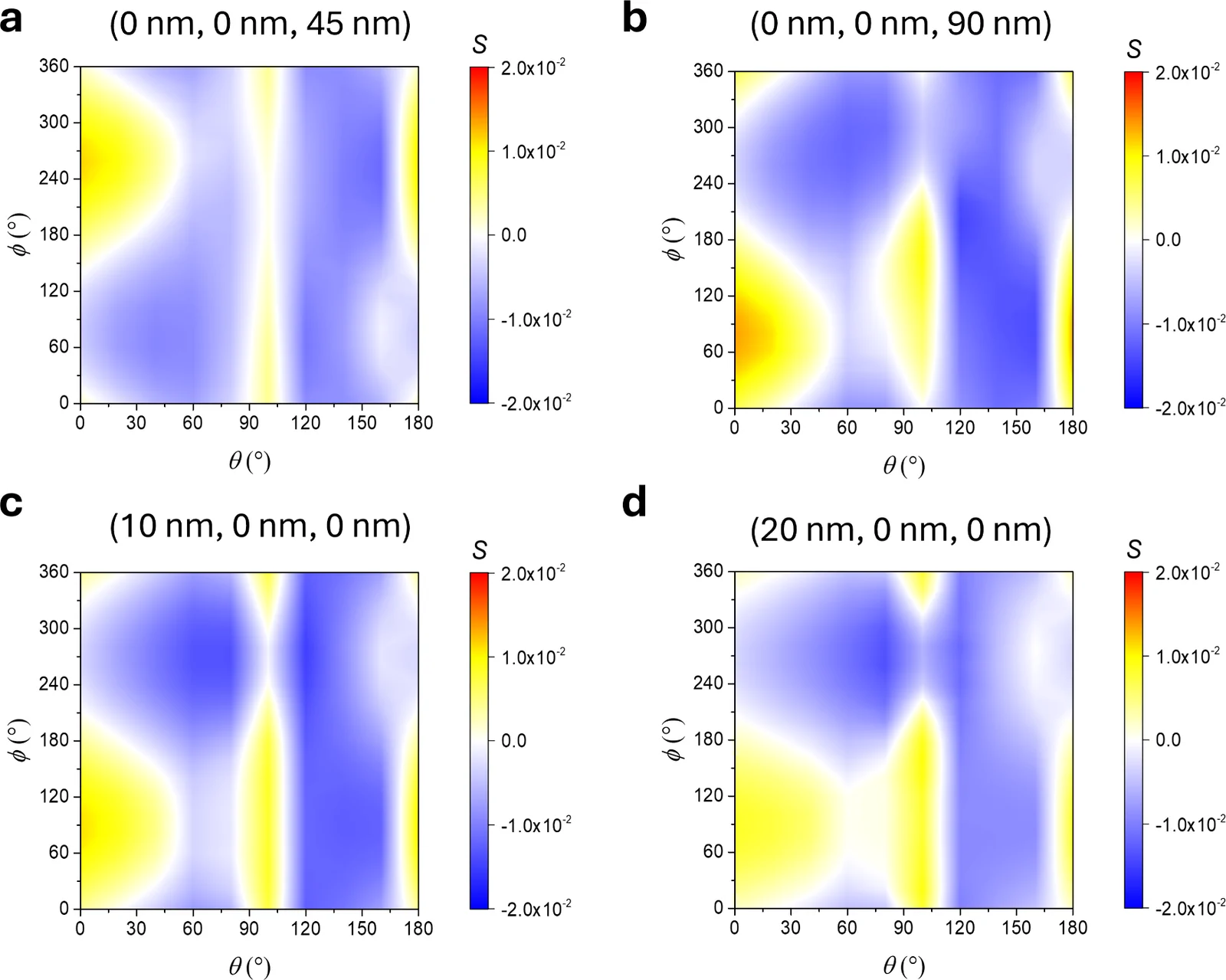
Spatial dependence of the *S*-orientation maps for the molecule located at (*x*,*y*,*z*) of (a) (0 nm, 0 nm, 45 nm), (b) (0 nm, 0 nm, 90 nm), (c) (10 nm, 0 nm, 0 nm), and (d) (20 nm, 0 nm, 0 nm). The origin is defined at the center of the helix.

The orientation-averaged values of *S* for Figure 4a and Figure 4b are −(7.6 ± 5.7) × 10^–3^ and −(8.4 ± 7.4) × 10^–3^, respectively, indicating that vertical displacement produces only modest variations in the overall enantioselectivity. This behavior likely reflects the helical periodicity of the structure. For the in-plane displacements shown in Figure 4c and Figure 4d, the averaged values decrease to −(5.7 ± 7.4) × 10^–3^ and −(3.0 ± 6.2) × 10^–3^, respectively, suggesting that in-plane displacement gradually weakens the enantioselective effect. Notably, this trend differs from the conventional picture in plasmonic nanostructures, where stronger light–matter interactions are typically achieved by reducing the molecule–nanostructure separation.^76,77^ In the present system, the optical modes supported by Mie resonances of the dielectric Si nanohelix are not strongly confined to the particle surface. Consequently, the chiral light–matter interaction extends over a larger volume, providing enhanced spatial robustness and representing a key advantage of dielectric nanophotonic platforms.

Our key findings can be summarized as follows. First, we demonstrate that chiral discrimination in radiative CT rates can be induced through weak chiral light–matter interactions. Second, we extend the mQED-ET theory by incorporating magnetic-field interactions, enabling the quantitative prediction of QED-driven chiral CT rates in arbitrary absorbing and dispersive media. Notably, both the helicene-based CT dyes and the Si nanohelix are experimentally realizable platforms,^68,70^ highlighting the practical feasibility of our theoretical framework. Third, we develop a numerical method to evaluate the term

Im[mCT·∇×Im⁡G®®(rM,rM,ω)·dCT*]

which is generally challenging to compute in realistic systems. Fourth, our theoretical framework predicts the robustness of chiral light–matter interactions for manipulating chiral discrimination. Specifically, the degree of discrimination reaches *S* = −2.0 × 10^–2^ for a particular molecular orientation and position, while orientation-averaged values remain on the order of 10^–3^ even when molecular isotropic rotations are taken into account. These results highlight the potential of QED-mediated chiral light–matter interactions as a strategy for controlling asymmetric radiative CT rates. Note that the present study is limited to chiral discrimination in CT rates, which is distinct from chemical enantioselectivity manifested as differences in the relative yields of enantiomeric products. To achieve chemical enantioselectivity based on this study, additional experimental design would be required to enable kinetic control of product formation,^78^ such that the asymmetric CT rate difference between the two enantiomers can be translated into enantiomeric product difference. In addition, chiral light–matter interactions do not necessarily rely on chiral photonic structures. Previous studies have demonstrated that weak light–matter coupling between achiral plasmonic/photonic materials and chiral molecules can enhance circular dichroism associated with molecular transitions,^79,80^ induce chiral plasmonic resonances^71^ or exhibit hierarchical chirality.^81^ Furthermore, the present mQED-ET framework is formulated in the single-molecule limit, whereas most experimental systems involve molecular ensembles. Consequently, the influence of collective effects on chiral discrimination remains an important topic.^82^ Assuming that the collective effect only considers the √*N* rate enhancements where *N* is the molecular number, it would not affect the chiral discrimination in our study since both *k*_ch_ and *k*_el_ exhibit the same *N* dependence, which therefore cancels out in the definition of *S*. However, this treatment represents only a rough approximation, as the conventional description of collective effects does not account for possible chiral energy-transfer processes between molecules.^65^ A rigorous treatment incorporating such intermolecular chiral interactions is beyond the scope of the present study and warrants further investigation. In summary, this work establishes a generalized theoretical framework for predicting chiral discrimination in radiative CT processes under weak light–matter coupling. We anticipate that these findings will stimulate further theoretical and experimental advances in molecular chiral discrimination mediated by QED effects.

## Computational Methods

### Quantum Chemical Calculation

We performed density functional theory (DFT) calculations at the B3LYP/ 6-31+g­(d) level with the Gaussian 16 program.^83^ The geometries of the ground state and excited states were optimized using DFT and time-dependent DFT (TD-DFT), respectively. The polarizable continuum method (PCM) model was used to simulate the solvent environment (cyclohexane).^84^

### Numerical Parameters for Evaluating *k*_el_ and *k*_ch_

We use eqs 4 and 5 as the working equations to evaluate *k*_ch_. To evaluate the local dyadic Green’s function its curl term, we applied the Lumerical FDTD Solutions^85^ to obtain the Purcell factors of the chiral dipoles simulated by an electric dipole and a magnetic dipole at the same position. According to eq 6, the polaritonic spectral density (eq S39) and the chiral spectral density (eq 5) can be obtained via summing and subtracting the Purcell factors of the chiral dipoles with the opposite sign in **d**_CT_. For the spectral integration, we follow our previous work to fulfill the long-wavelength approximation.^53,57,60^ In this study, the upper bound and the lower bound frequencies are set as 4.13 and 1.77 eV for all systems. The dielectric functions of silicon from Schinke was used for the Si nanohelix.^86^ The simulation domain is a cube with a side length of 1.5 μm. The spatial mesh sizes are 2 nm along the *x* and *y* directions and 3 nm along the *z* direction. The autoshutoff threshold is set to 10^–6^.

On the other hand, the parameters of ρ_vib_(ω) under the limit of low vibrational frequency include the energy gap Δ*E*, temperature *T* and reorganization energy *E*_r_, which can be obtained from the reported experimental results.^68^ Δ*E* = 2.84 eV corresponds to the peak energy of the steady-state emission spectra. *T* = 300 K represents room temperature. *E*_r_ = 0.13 eV can be estimated from the Stokes shift of the steady-state spectra.^87,88^

## Supplementary Material



## Notes

Model Hamiltonian; small-polaron transformation; derivation of chiral CT rate; characterization of numerical accuracy; orientation dependence of *k*_el_ and *k*_ch_; spectral dependence of *J*_ch_/*J*_el_; cross sections of the system in the *xy* plane (*z* = 0); supporting references (PDF)
