# Supplementary material for: Inducing Enantioselectivity in Radiative Charge-Transfer Rates through Chiral Quantum Electrodynamic Fluctuations
Source: J Phys Chem Lett. 2026 Oct 5;17(41):11836–44. doi: 10.1021/acs.jpclett.6c02328 (PMC13679079; doi:10.1021/acs.jpclett.6c02328)
Supplement: Supplementary file 1 [file jz6c02328_si_001.pdf]

Supporting Information: Inducing Enantioselectivity in Radiative Charge-Transfer Rates through Chiral Quantum Electrodynamic Fluctuations

Yu-Chen Wei[*,†] and Liang-Yan Hsu[‡,¶,§]

†*Department of Chemistry, National Tsing Hua University, Hsinchu 300044, Taiwan*

‡*Institute of Atomic and Molecular Sciences, Academia Sinica, Taipei 106, Taiwan*

¶*Department of Chemistry, National Taiwan University, Taipei 106, Taiwan*

§*National Center for Theoretical Sciences, Taipei 106, Taiwan*

E-mail: yc.wei@mx.nthu.edu.tw

Contents

S1 Model Hamiltonian **S3**

S2 Small-Polaron Transformation **S6**

S3 Derivation of Chiral CT Rate **S7**

Supporting Figures **S12**

Supplementary References **S16**

S1 Model Hamiltonian

According to the previous study,[1,2] the first two terms in Eq. 1 in the manuscript correspond to the standard model of molecular electron transfer (ET). As a result, these two terms can be simplified as a two-state molecular model in the electronic subspace with a harmonic vibrational bath. as shown in Eqs. S2–S4. In this framework, we regroup the terms in Eq. 1 in the manuscript as $\hat{H}_{\text{ele}}$−the electronic Hamiltonian, $\hat{H}_{\text{vib}}$−the vibrational Hamiltonian, $\hat{H}_{\text{ele-vib}}$−the electron-vibrational (vibronic) coupling, $\hat{H}_{\text{pol}}$−the polaritonic Hamiltonian and $\hat{H}_{\text{mol-pol}}$− the light-molecule interaction in the following,

$$\hat{H} = \hat{H}_{\text{ele}} + \hat{H}_{\text{vib}} + \hat{H}_{\text{pol}} + \hat{H}_{\text{ele-vib}} + \hat{H}_{\text{mol-pol}}. \tag{S1}$$

$$\hat{H}_{\text{ele}} = E_{\text{DA}} \left|\text{DA}\right\rangle \left\langle\text{DA}\right| + E_{\text{D}^+\text{A}^-} \left|\text{D}^+\text{A}^-\right\rangle \left\langle\text{D}^+\text{A}^-\right| + V_{\text{CT}}(\left|\text{DA}\right\rangle \left\langle\text{D}^+\text{A}^-\right| + \text{h.c.}). \tag{S2}$$

$$\hat{H}_{\text{vib}} = \sum_{u=1}^{m_{\text{vib}}} \hbar\omega_{\text{vib},u} \hat{b}_u^\dagger \hat{b}_u. \tag{S3}$$

$$\hat{H}_{\text{ele-vib}} = \sum_{u=1}^{m_{\text{vib}}} \hbar\omega_{\text{vib},u} \left[\sqrt{S_{\text{vib},u}}(\hat{b}_u^\dagger + \hat{b}_u) + S_{\text{vib},u})\right] \left|\text{D}^+\text{A}^-\right\rangle \left\langle\text{D}^+\text{A}^-\right|. \tag{S4}$$

$$\hat{H}_{\text{pol}} = \int d\mathbf{r} \int_0^\infty d\omega \hbar\omega \hat{\mathbf{f}}^\dagger(\mathbf{r},\omega) \cdot \hat{\mathbf{f}}(\mathbf{r},\omega). \tag{S5}$$

$$\hat{H}_{\text{mol-pol}} = -\hat{\boldsymbol{\mu}} \cdot \hat{\mathbf{E}}(\mathbf{r}_{\text{M}}) - \hat{\mathbf{m}} \cdot \hat{\mathbf{B}}(\mathbf{r}_{\text{M}}). \tag{S6}$$

Here, the symbol D denotes electron-donor moiety, and the symbol A denotes electron-acceptor moiety; the symbol D^+ stands for the donor moiety losing an electron, and the symbol A^- stands for the acceptor moiety obtaining an electron. E_{DA} and $E_{\text{D}^+\text{A}^-}$ correspond to the energies of the initial and final states, respectively. V_{CT} is the Coulomb electronic coupling. The vibrational Hamiltonian $\hat{H}_{\text{vib}}$ is modeled as m_{vib} harmonic vibrational modes, and the electron-vibrational coupling $\hat{H}_{\text{ele-vib}}$ is modeled as a linear coupling between vibrational modes and the electronic state. The uth vibrational mode possesses the frequency $\omega_{\text{vib},u}$ with the vibrational creation (annihilation) operator $\hat{b}_u^\dagger(\hat{b}_u)$ and the vibrational Huang-Rhys factor $S_{\text{vib},u}$. $\mathbf{r}_{\text{M}}$ is the position of the molecular center.

Next, the definition of electric/magnetic field operators in the macroscopic QED are shown below,[3–5]

$$
\begin{aligned}
\hat{\mathbf{E}}(\mathbf{r}) &= \int_0^\infty d\omega \underline{\hat{\mathbf{E}}}(\mathbf{r},\omega) + \text{h.c.} \\
&= \sum_{\lambda=e,m} \int_0^\infty d\omega \int d\mathbf{r}' \; \overline{\overline{\mathbf{G}}}_\lambda(\mathbf{r},\mathbf{r}',\omega) \cdot \hat{\mathbf{f}}_\lambda(\mathbf{r}',\omega) + \text{h.c.},
\end{aligned} \tag{S7}
$$

$$
\begin{aligned}
\hat{\mathbf{B}}(\mathbf{r}) &= \int_0^\infty d\omega \underline{\hat{\mathbf{B}}}(\mathbf{r},\omega) + \text{h.c.} \\
&= \sum_{\lambda=e,m} \int_0^\infty \frac{d\omega}{i\omega} \int d\mathbf{r}' \; \nabla \times \overline{\overline{\mathbf{G}}}_\lambda(\mathbf{r},\mathbf{r}',\omega) \cdot \hat{\mathbf{f}}_\lambda(\mathbf{r}',\omega) + \text{h.c.}.
\end{aligned} \tag{S8}
$$

Here $\underline{\hat{\mathbf{E}}}(\mathbf{r},\omega)$ and $\underline{\hat{\mathbf{B}}}(\mathbf{r},\omega)$ are the frequency components of the electric field $\hat{\mathbf{E}}(\mathbf{r})$ and magnetic field $\hat{\mathbf{B}}(\mathbf{r})$, respectively. $\lambda = e/m$ indicates electric/magnetic type. The creation and annihilation operators $\hat{\mathbf{f}}_\lambda(\mathbf{r},\omega)$ fulfill the commutation relation $[\hat{\mathbf{f}}_\lambda(\mathbf{r},\omega), \hat{\mathbf{f}}^\dagger_{\lambda'}(\mathbf{r}',\omega')] = \delta_{\lambda\lambda'}\delta(\mathbf{r}-\mathbf{r}')\delta(\omega-\omega')$. The dyadic Green's function $\overline{\overline{\mathbf{G}}}_\lambda(\mathbf{r}_\mathrm{M},\mathbf{r},\omega)$ is defined via the Helmholtz equation and is given by

$$
\overline{\overline{\mathbf{G}}}_e(\mathbf{r},\mathbf{r}',\omega) = i\frac{\omega^2}{c^2}\sqrt{\frac{\hbar}{\pi\epsilon_0}\mathrm{Im}\epsilon_\mathrm{r}(\mathbf{r}',\omega)}\; \overline{\overline{\mathbf{G}}}(\mathbf{r},\mathbf{r}',\omega), \tag{S9}
$$

$$
\overline{\overline{\mathbf{G}}}_m(\mathbf{r},\mathbf{r}',\omega) = i\frac{\omega}{c}\sqrt{\frac{\hbar}{\pi\epsilon_0}\frac{\mathrm{Im}\mu_\mathrm{r}(\mathbf{r}',\omega)}{|\mu_\mathrm{r}(\mathbf{r}',\omega)|^2}}\; \overline{\overline{\mathbf{G}}}(\mathbf{r},\mathbf{r}',\omega) \times \overleftarrow{\nabla}', \tag{S10}
$$

where ϵ_r and μ_r are the relative permittivity and the relative permeability, respectively. The operation $\times\overleftarrow{\nabla}$ indicates that the derivatives acts on the second spatial variable.[5,6]

In addition, Eqs. S7 and S8 can also be expressed as the response of the polarization field

$\hat{\underline{\mathbf{P}}}(\mathbf{r},\omega)$ and the magnetism field $\hat{\underline{\mathbf{M}}}(\mathbf{r},\omega)$.[5,7]

$$\begin{aligned}\hat{\underline{\mathbf{E}}}(\mathbf{r},\omega) =& \frac{\omega^2}{\epsilon_0 c^2}\int d\mathbf{r}'\ \overline{\overline{\mathbf{G}}}_\lambda(\mathbf{r},\mathbf{r}',\omega)\cdot\hat{\underline{\mathbf{P}}}(\mathbf{r}',\omega)\\ &- i\mu_0\omega\int d\mathbf{r}'\ [\overline{\overline{\mathbf{G}}}(\mathbf{r},\mathbf{r}',\omega)\times\overleftarrow{\nabla}']\cdot\hat{\underline{\mathbf{M}}}(\mathbf{r}',\omega),\end{aligned} \tag{S11}$$

$$\begin{aligned}\hat{\underline{\mathbf{B}}}(\mathbf{r},\omega) =& i\mu_0\omega\int d\mathbf{r}'\ [\nabla\times\overline{\overline{\mathbf{G}}}(\mathbf{r},\mathbf{r}',\omega)]\cdot\hat{\underline{\mathbf{P}}}(\mathbf{r}',\omega)\\ &- \mu_0\int d\mathbf{r}'\ [\nabla\times\overline{\overline{\mathbf{G}}}(\mathbf{r},\mathbf{r}',\omega)\times\overleftarrow{\nabla}']\cdot\hat{\underline{\mathbf{M}}}(\mathbf{r}',\omega).\end{aligned} \tag{S12}$$

Considering that a single chiral molecule is at $\mathbf{r}_\mathrm{M}$ and the molecular size is much smaller than the optical wavelength, it is reasonable to make the point-dipole approximation and assume that $\hat{\underline{\mathbf{P}}}(\mathbf{r},\omega) = \delta(\mathbf{r}-\mathbf{r}_\mathrm{M})\hat{\underline{\mathbf{p}}}(\omega)$ and $\hat{\underline{\mathbf{M}}}(\mathbf{r},\omega) = \delta(\mathbf{r}-\mathbf{r}_\mathrm{M})\hat{\underline{\mathbf{m}}}(\omega)$.[8] The approximation leads to

$$\begin{aligned}\hat{\underline{\mathbf{E}}}(\mathbf{r},\omega) =& \frac{\omega^2}{\epsilon_0 c^2}\overline{\overline{\mathbf{G}}}_\lambda(\mathbf{r},\mathbf{r}_\mathrm{M},\omega)\cdot\hat{\underline{\mathbf{p}}}(\omega)\\ &- i\mu_0\omega[\overline{\overline{\mathbf{G}}}(\mathbf{r},\mathbf{r}_\mathrm{M},\omega)\times\overleftarrow{\nabla}']\cdot\hat{\underline{\mathbf{m}}}(\mathbf{r}_\mathrm{M},\omega),\end{aligned} \tag{S13}$$

$$\begin{aligned}\hat{\underline{\mathbf{B}}}(\mathbf{r},\omega) =& i\mu_0\omega[\nabla\times\overline{\overline{\mathbf{G}}}(\mathbf{r},\mathbf{r}_\mathrm{M},\omega)]\cdot\hat{\underline{\mathbf{p}}}(\omega)\\ &- \mu_0[\nabla\times\overline{\overline{\mathbf{G}}}(\mathbf{r},\mathbf{r}_\mathrm{M},\omega)\times\overleftarrow{\nabla}']\cdot\hat{\underline{\mathbf{m}}}(\omega).\end{aligned} \tag{S14}$$

Eqs. S13 and S14 demonstrate how a chiral molecule generate electric and magnetic fields through the dyadic Green's function and its derivatives.

To derive the light-matter coupling strength, it is necessary to derive the matrix elements of the $\hat{H}_\mathrm{mol-pol}$ in the electronic subspace. Within the Condon approximation, i.e., the nuclear degrees of freedom do not involve light-molecule interaction, one can derive the charge transfer electric dipole moment and the charge transfer magnetic dipole moment as

$$\begin{aligned}\mathbf{d}_\mathrm{CT} &\equiv \langle \mathrm{DA}|\,\hat{\boldsymbol{\mu}}\,|\mathrm{D^+A^-}\rangle\,,\\ \mathbf{m}_\mathrm{CT} &\equiv \langle \mathrm{DA}|\,\hat{\mathbf{m}}\,|\mathrm{D^+A^-}\rangle\,.\end{aligned} \tag{S15}$$

For chiral molecules, $\mathbf{d}_{\mathrm{CT}}$, a polar vector, reverses sign while $\mathbf{m}_{\mathrm{CT}}$, an axial vector, remains unchanged under reflection.[9]

Based on Eq. S15, we can rewrite $\hat{H}_{\text{mol-pol}}$ as

$$\hat{H}_{\text{mol-pol}} = -(\mathbf{d}_{\mathrm{CT}} \cdot \hat{\mathbf{E}}(\mathbf{r}_{\mathrm{M}}) + \mathbf{m}_{\mathrm{CT}} \cdot \hat{\mathbf{B}}(\mathbf{r}_{\mathrm{M}})) \left|\mathrm{DA}\right\rangle \left\langle \mathrm{D^{+}A^{-}}\right| - \text{h.c.} \tag{S16}$$

S2 Small-Polaron Transformation

Here we apply the unitary transformation along the vibrational basis via[10,11]

$$\hat{U}_{\mathrm{vib}} \equiv \exp\left\{ \sum_{u=1}^{m_{\mathrm{vib}}} \sqrt{S_{\mathrm{vib},u}} (\hat{b}_u^{\dagger} - \hat{b}_u)) \left|\mathrm{D^{+}A^{-}}\right\rangle \left\langle \mathrm{D^{+}A^{-}}\right| \right\}, \tag{S17}$$

The transformed Hamiltonian can be written as follows.

$$\hat{\mathscr{H}} \equiv \hat{U}_{\mathrm{vib}} \hat{H} \hat{U}_{\mathrm{vib}}^{\dagger} = \hat{\mathscr{H}}_{\mathrm{ele}} + \hat{\mathscr{H}}_{\mathrm{vib}} + \hat{\mathscr{H}}_{\mathrm{pol}} + \hat{\mathscr{H}}_{\mathrm{mol-pol}}, \tag{S18}$$

$$\hat{\mathscr{H}}_{\mathrm{ele}} \approx E_{\mathrm{DA}} \left|\mathrm{DA}\right\rangle \left\langle \mathrm{DA}\right| + E_{\mathrm{D^{+}A^{-}}} \left|\mathrm{D^{+}A^{-}}\right\rangle \left\langle \mathrm{D^{+}A^{-}}\right| + V_{\mathrm{CT}} (\left|\mathrm{DA}\right\rangle \left\langle \mathrm{D^{+}A^{-}}\right| \hat{D}_{\mathrm{vib}} + \text{h.c.}), \tag{S19}$$

$$\hat{\mathscr{H}}_{\mathrm{vib}} = \sum_{u=1}^{m_{\mathrm{vib}}} \hbar\omega_{\mathrm{vib},u} \hat{b}_u^{\dagger} \hat{b}_u, \tag{S20}$$

$$\hat{\mathscr{H}}_{\mathrm{pol}} = \int d\mathbf{r} \int_0^{\infty} d\omega\ \hbar\omega \hat{\mathbf{f}}^{\dagger}(\mathbf{r},\omega) \cdot \hat{\mathbf{f}}(\mathbf{r},\omega), \tag{S21}$$

$$\hat{\mathscr{H}}_{\mathrm{mol-pol}} = -(\mathbf{d}_{\mathrm{CT}} \cdot \hat{\mathbf{E}}(\mathbf{r}_{\mathrm{M}}) + \mathbf{m}_{\mathrm{CT}} \cdot \hat{\mathbf{B}}(\mathbf{r}_{\mathrm{M}})) \left|\mathrm{DA}\right\rangle \left\langle \mathrm{D^{+}A^{-}}\right| \hat{D}_{\mathrm{vib}} + \text{h.c.}, \tag{S22}$$

where $\hat{D}_{\mathrm{vib}} = \exp\left\{\sum_{u=1}^{m_{\mathrm{vib}}} \sqrt{S_{\mathrm{vib},u}} (\hat{b}_u^{\dagger} - \hat{b}_u)\right\}$. According to Eqs. S19 and S22, we can define the new effective ET coupling $\hat{\mathscr{V}}_{\mathrm{CT}}$ by collecting all the electronic off-diagonal terms together.

$$\hat{\mathscr{V}}_{\mathrm{CT}} \equiv \left(\mathrm{V}_{\mathrm{ET}} - \mathbf{d}_{\mathrm{CT}} \cdot \hat{\mathbf{E}}(\mathbf{r}_{\mathrm{M}}) - \mathbf{m}_{\mathrm{CT}} \cdot \hat{\mathbf{B}}(\mathbf{r}_{\mathrm{M}})\right) \left|\mathrm{DA}\right\rangle \left\langle \mathrm{D^{+}A^{-}}\right| \hat{D}_{\mathrm{vib}} + \text{h.c.}. \tag{S23}$$

S3 Derivation of Chiral ET Rate

First, we denote $\{M\} \equiv \{M_1, \ldots, M_u, \ldots, M_{m_{\text{vib}}}\}$ and $\{M'\}$ the initial and final states of the vibrational basis. Note that the notation ($'$) represents the basis of the final state. Based on this setting and the Fermi's golden rule, the total ET rate can be expressed as follows.

$$k_{\text{CT}} = k_{\text{Marcus}} + k_{\text{QED}}, \tag{S24}$$

$$k_{\text{Marcus}} = \frac{2\pi}{\hbar} \sum_{\{M\},\{M'\}} \mathrm{P}_{\{M\}} |\langle \mathrm{DA}, \{M\}, \{0\}| \hat{\mathcal{V}}_{\text{CT}} |\mathrm{D^+A^-}, \{M'\}, \{0\}\rangle|^2 \times \delta(E_{\mathrm{DA},\{M\},\{0\}} - E_{\mathrm{D^+A^-},\{M'\},\{0\}}), \tag{S25}$$

$$\begin{aligned} k_{\text{QED}} &= \int d\mathbf{r} \int_0^\infty d\omega\ \kappa_{\text{CT},\{0\}\to\{\mathbf{1}(\mathbf{r},\omega)\}} \\ &= \frac{2\pi}{\hbar} \sum_{\{M\},\{M'\}} \mathrm{P}_{\{M\}} \int d\mathbf{r} \int_0^\infty d\omega\ |\langle \mathrm{DA}, \{M\}, \{0\}| \hat{\mathcal{V}}_{\text{CT}} |\mathrm{D^+A^-}, \{M'\}, \{\mathbf{1}(\mathbf{r},\omega)\}\rangle|^2 \\ &\quad \times \delta(E_{\mathrm{DA},\{M\},\{0\}} - E_{\mathrm{D^+A^-},\{M'\},\{\mathbf{1}(\mathbf{r},\omega)\}}). \end{aligned} \tag{S26}$$

$\mathrm{P}_{\{M\}}$ is the population distribution that characterizes the vibrational bath. $|\mathrm{D^+A^-}, \{M'\}, \{0\}\rangle = |\mathrm{D^+A^-}\rangle \otimes |\{M'\}\rangle \otimes \{0\}$ stands for a Fock state which describes the final electronic state $|\mathrm{D^+A^-}\rangle$ combined with the vibrational state $|\{M'\}\rangle$ and a polaritonic state $\{0\}$. Here, $\{0\}$ and $\{\mathbf{1}(\mathbf{r},\omega)\}$ indicate the vacuum polaritonic state and the single polaritonic state with the frequency ω monitored at the position $\mathbf{r}$, respectively. Note that we neglect the temperature effect on the photon population and assume that the initial population of photons is fully at the polariton vacuum state.[12,13]

In addition, we can use the similar approach to solve $\kappa_{\text{CT},\{0\}\to\{\mathbf{1}(\mathbf{r},\omega)\}}$. Starting from the Fermi's golden rule,

$$k_{\text{QED}} = \frac{1}{\hbar^2} \int d\mathbf{r} \int_0^\infty d\omega \int_{-\infty}^\infty \mathrm{d}t\ \exp\{\frac{-i(\Delta\mathrm{E} + \hbar\omega)t}{\hbar}\} \langle \hat{\mathcal{V}}^{0,\mathbf{1}(\mathbf{r},\omega)}_{\mathrm{DA,D^+A^-}}(t) \hat{\mathcal{V}}^{\mathbf{1}(\mathbf{r},\omega),0}_{\mathrm{D^+A^-,DA}} \rangle. \tag{S27}$$

where

$$\hat{\mathscr{V}}^{0,\mathbf{1}(\mathbf{r},\omega)}_{\mathrm{D^+A^-},\mathrm{DA}} = \langle \mathrm{D^+A^-}, \{0\}| \,\hat{\mathscr{V}}_{\mathrm{CT}}\, |\mathrm{DA}, \{\mathbf{1}(\mathbf{r},\omega)\}\rangle$$
$$= -\langle\{0\}|\,(\mathbf{d}_{\mathrm{CT}}\cdot\hat{\mathbf{E}}(\mathbf{r}_{\mathrm{M}}) + \mathbf{m}_{\mathrm{CT}}\cdot\hat{\mathbf{B}}(\mathbf{r}_{\mathrm{M}}))\,|\{\mathbf{1}(\mathbf{r},\omega)\}\rangle\,\hat{D}_{\mathrm{vib}}. \tag{S28}$$

Based on Eq. (S28), the formalism of $\kappa_{\mathrm{CT},0\to 1_{n'}}$ can be expressed as

$$\begin{aligned}
&k_{\mathrm{QED}}\\
&= \frac{1}{\hbar^2}\int d\mathbf{r}\int_0^\infty d\omega |\,\langle\{0\}|\,\mathbf{d}_{\mathrm{CT}}\cdot\hat{\mathbf{E}}(\mathbf{r}_{\mathrm{M}}) + \mathbf{m}_{\mathrm{CT}}\cdot\hat{\mathbf{B}}(\mathbf{r}_{\mathrm{M}})\,|\{\mathbf{1}(\mathbf{r},\omega)\}\rangle\,|^2\\
&\times \exp\Big\{\sum_{u=1}^{m_{\mathrm{vib}}} -(2M_u+1)S_{\mathrm{vib},u}\Big\}\int_{-\infty}^{\infty}\mathrm{d}t\exp\{\frac{-i(\Delta\mathrm{E}+\hbar\omega)}{\hbar}\}\\
&\times \exp\Big\{\sum_{u=1}^{m_{\mathrm{vib}}}(M_u+1)S_{\mathrm{vib},u}\exp\big\{-i\omega_{\mathrm{vib},u}t\big\} + M_u S_{\mathrm{vib},u}\exp\big\{i\omega_{\mathrm{vib},u}t\big\}\Big\}\\
&= \frac{2\pi}{\hbar}\int d\mathbf{r}\int_0^\infty d\omega |\,\langle\{0\}|\,\mathbf{d}_{\mathrm{CT}}\cdot\hat{\mathbf{E}}(\mathbf{r}_{\mathrm{M}}) + \mathbf{m}_{\mathrm{CT}}\cdot\hat{\mathbf{B}}(\mathbf{r}_{\mathrm{M}})\,|\{\mathbf{1}(\mathbf{r},\omega)\}\rangle\,|^2\rho_{\mathrm{vib}}(\omega),
\end{aligned} \tag{S29}$$

where $\rho_{\mathrm{vib}}(\omega)$, dubbed vibronic density of states (DOS) or vibrational transition DOS,[13] corresponds to the DOS of molecular degrees of freedoms, and it is defined as

$$\begin{aligned}
\rho_{\mathrm{vib}}(\omega) \equiv\ & \frac{1}{2\pi\hbar}\exp\Big\{\sum_{u=1}^{m_{\mathrm{vib}}} -(2M_u+1)S_{\mathrm{vib},u}\Big\}\int_{-\infty}^{\infty}\mathrm{d}t\,\exp\{\frac{-i(\Delta E+\hbar\omega)t}{\hbar}\}\\
&\times \exp\Big\{\sum_{u=1}^{m_{\mathrm{vib}}}(M_u+1)S_{\mathrm{vib},u}\exp\big\{-i\omega_{\mathrm{vib},u}t\big\} + M_u S_{\mathrm{vib},u}\exp\big\{i\omega_{\mathrm{vib},u}t\big\}\Big\},
\end{aligned} \tag{S30}$$

which is as same as the definition in the QED-ET theory.[13] Next, the coupling term in Eq. S29 can be expanded as

$$\begin{aligned}
&\langle\{0\}|\,\mathbf{d}_{\mathrm{CT}}\cdot\hat{\mathbf{E}}(\mathbf{r}_{\mathrm{M}}) + \mathbf{m}_{\mathrm{CT}}\cdot\hat{\mathbf{B}}(\mathbf{r}_{\mathrm{M}})\,|\{\mathbf{1}(\mathbf{r},\omega)\}\rangle\\
&= \sum_{\lambda=e,m}\big[\int d\mathbf{r}'\int_0^\infty d\omega'\mathbf{d}_{\mathrm{CT}}\cdot\overline{\overline{\mathbf{G}}}_\lambda(\mathbf{r}_{\mathrm{M}},\mathbf{r}',\omega') + \frac{1}{i\omega'}\mathbf{m}_{\mathrm{CT}}\cdot\nabla\times\overline{\overline{\mathbf{G}}}_\lambda(\mathbf{r}_{\mathrm{M}},\mathbf{r}',\omega')\big]\delta(\mathbf{r}-\mathbf{r}')\delta(\omega-\omega')\\
&= \sum_{\lambda=e,m}\big[\mathbf{d}_{\mathrm{CT}}\cdot\overline{\overline{\mathbf{G}}}_\lambda(\mathbf{r}_{\mathrm{M}},\mathbf{r},\omega) + \frac{1}{i\omega}\mathbf{m}_{\mathrm{CT}}\cdot\nabla\times\overline{\overline{\mathbf{G}}}_\lambda(\mathbf{r}_{\mathrm{M}},\mathbf{r},\omega)\big].
\end{aligned} \tag{S31}$$

At this point we calculate the square of coupling strength employing Eq. S31 and integrate over the space $\mathbf{r}$. We hereby obtain a purely electric term responsible for the electric contribution of QED-ET processes as follows.

$$\sum_{\lambda=e,m}\int d\mathbf{r}\ |\mathbf{d}_{\mathrm{CT}}\cdot\overline{\overline{\mathbf{G}}}_{\lambda}(\mathbf{r}_{\mathrm{M}},\mathbf{r},\omega)|^2 \\ =\frac{\hbar\mu_0}{\pi}\omega^2\mathbf{d}_{\mathrm{CT}}\cdot\mathrm{Im}\overline{\overline{\mathbf{G}}}(\mathbf{r}_{\mathrm{M}},\mathbf{r}_{\mathrm{M}},\omega)\cdot\mathbf{d}^*_{\mathrm{CT}}. \tag{S32}$$

Here we use the integral relation of the dyadic Green's function.[5,14]

$$\sum_{\lambda=e,m}\int d\mathbf{r}\ \overline{\overline{\mathbf{G}}}_{\lambda}(\mathbf{r}_{\alpha},\mathbf{r},\omega)\cdot\overline{\overline{\mathbf{G}}}^*_{\lambda}(\mathbf{r},\mathbf{r}_{\beta},\omega)=\frac{\hbar\mu_0}{\pi}\omega^2\mathrm{Im}\overline{\overline{\mathbf{G}}}(\mathbf{r}_{\alpha},\mathbf{r}_{\beta},\omega). \tag{S33}$$

Then there is a purely magnetic term, which we will neglect due to the lower magnitude of the magnetic transition dipole compared with the electric transition. dipole. Finally we have cross-terms, which are responsible for driving force of chiral ET. These have the following structure:

$$\frac{1}{i\omega}\sum_{\lambda=e,m}\int d\mathbf{r}\ \left[\mathbf{d}_{\mathrm{CT}}\cdot\overline{\overline{\mathbf{G}}}_{\lambda}(\mathbf{r}_{\mathrm{M}},\mathbf{r},\omega)\cdot\overline{\overline{\mathbf{G}}}^*_{\lambda}(\mathbf{r},\mathbf{r}_{\mathrm{M}},\omega)\times\overleftarrow{\nabla}\cdot\mathbf{m}^*_{\mathrm{CT}}+\mathrm{h.c.}\right] \\ =-\frac{1}{i\omega}\frac{\hbar\mu_0}{\pi}\omega^2\left[\mathbf{d}_{\mathrm{CT}}\cdot\mathrm{Im}\overline{\overline{\mathbf{G}}}(\mathbf{r}_{\mathrm{M}},\mathbf{r}_{\mathrm{M}},\omega)\times\overleftarrow{\nabla}\cdot\mathbf{m}^*_{\mathrm{CT}}-\mathrm{h.c.}\right]. \tag{S34}$$

By exploiting the Onsager reciprocity of the Green's tensor and the identity of the Levi-Civita symbol, we obtain this relation

$$\mathbf{d}_{\mathrm{CT}}\cdot\mathrm{Im}\overline{\overline{\mathbf{G}}}(\mathbf{r}_{\mathrm{M}},\mathbf{r}_{\mathrm{M}},\omega)\times\overleftarrow{\nabla}\cdot\mathbf{m}^*_{\mathrm{CT}}=\left[\mathbf{m}_{\mathrm{CT}}\cdot\nabla\times\mathrm{Im}\overline{\overline{\mathbf{G}}}(\mathbf{r}_{\mathrm{M}},\mathbf{r}_{\mathrm{M}},\omega)\cdot\mathbf{d}^*_{\mathrm{CT}}\right]^*. \tag{S35}$$

Based on Eq. S35, Eq. S34 becomes

$$
\begin{aligned}
& -\frac{1}{i\omega}\sum_{\lambda=e,m}\int d\mathbf{r}\ \big[\mathbf{d}_{\mathrm{CT}}\cdot\overline{\overline{\mathbf{G}}}_{\lambda}(\mathbf{r}_{\mathrm{M}},\mathbf{r},\omega)\cdot\overline{\overline{\mathbf{G}}}^{*}_{\lambda}(\mathbf{r},\mathbf{r}_{\mathrm{M}},\omega)\times\overleftarrow{\nabla}\cdot\mathbf{m}^{*}_{\mathrm{CT}}-\mathrm{h.c.}\big] \\
& = -\frac{1}{i\omega}\frac{\hbar\mu_0}{\pi}\omega^2\big[\mathbf{d}_{\mathrm{CT}}\cdot\mathrm{Im}\overline{\overline{\mathbf{G}}}(\mathbf{r}_{\mathrm{M}},\mathbf{r}_{\mathrm{M}},\omega)\times\overleftarrow{\nabla}\cdot\mathbf{m}^{*}_{\mathrm{CT}}-\mathrm{h.c.}\big] \\
& = \frac{2\hbar\mu_0}{\pi}\omega\ \mathrm{Im}\big[\mathbf{m}_{\mathrm{CT}}\cdot\nabla\times\mathrm{Im}\overline{\overline{\mathbf{G}}}(\mathbf{r}_{\mathrm{M}},\mathbf{r}_{\mathrm{M}},\omega)\cdot\mathbf{d}^{*}_{\mathrm{CT}}\big]. \qquad \text{(S36)}
\end{aligned}
$$

According to Eq. S32 and S36, k_{QED} is derived as follows.

$$
k_{\mathrm{QED}} = \frac{2\pi}{\hbar}\int_0^{\infty} d\omega\frac{\hbar\mu_0}{\pi}\big\{\omega^2\mathbf{d}_{\mathrm{CT}}\cdot\mathrm{Im}\overline{\overline{\mathbf{G}}}(\mathbf{r}_{\mathrm{M}},\mathbf{r}_{\mathrm{M}},\omega)\cdot\mathbf{d}^{*}_{\mathrm{CT}} \qquad \text{(S37)}
$$

$$
+\,2\omega\ \mathrm{Im}\big[\mathbf{m}_{\mathrm{CT}}\cdot\nabla\times\mathrm{Im}\overline{\overline{\mathbf{G}}}(\mathbf{r}_{\mathrm{M}},\mathbf{r}_{\mathrm{M}},\omega)\cdot\mathbf{d}^{*}_{\mathrm{CT}}\big]\big\}\rho_{\mathrm{vib}}(\omega)
$$

$$
= \frac{2\pi}{\hbar}\int_0^{\infty} d\omega\ J_{\mathrm{pol}}(\omega)\rho_{\mathrm{vib}}(\omega), \qquad \text{(S38)}
$$

$J_{\mathrm{pol}}(\omega)$ corresponds to the polaritonic spectral density considering the chiral effect, and it is defined as

$$
J_{\mathrm{pol}}(\omega) \equiv \frac{\hbar\mu_0}{\pi}\big\{\omega^2\mathbf{d}_{\mathrm{CT}}\cdot\mathrm{Im}\overline{\overline{\mathbf{G}}}(\mathbf{r}_{\mathrm{M}},\mathbf{r}_{\mathrm{M}},\omega)\cdot\mathbf{d}^{*}_{\mathrm{CT}}+2\omega\ \mathrm{Im}\big[\mathbf{m}_{\mathrm{CT}}\cdot\nabla\times\mathrm{Im}\overline{\overline{\mathbf{G}}}(\mathbf{r}_{\mathrm{M}},\mathbf{r}_{\mathrm{M}},\omega)\cdot\mathbf{d}^{*}_{\mathrm{CT}}\big]\big\}, \qquad \text{(S39)}
$$

The first term in Eq. S39 leads to the radiative CT caused by the transition electric dipole moment only, which is consistent with the mQED-ET theory.[15] The second term in Eq. S39 causes the chiral CT arising from the cross-terms of the transition electric dipole moment and the transition magnetic dipole moment interacting with chiral polaritonic modes. This chiral CT component agrees with the spontaneous emission in chiral environment (chiral Purcell effect).[6] As a result, the total k_{QED} can be defined as the sum of electric and chiral components

$$
k_{\mathrm{QED}} = k_{\mathrm{el}} + k_{\mathrm{ch}}, \qquad \text{(S40)}
$$

where the electric component arises from the the first term in the polaritonic spectral density $J_{\rm el}(\omega)$

$$\begin{aligned} k_{\rm el} =& \frac{2\pi}{\hbar}\int_0^\infty d\omega\ J_{\rm el}(\omega)\rho_{\rm vib}(\omega) \\ =& 2\mu_0\int_0^\infty d\omega\ \omega^2 \mathbf{d}_{\rm CT}\cdot{\rm Im}\overline{\overline{\mathbf{G}}}(\mathbf{r}_{\rm M},\mathbf{r}_{\rm M},\omega)\cdot\mathbf{d}^*_{\rm CT}\rho_{\rm vib}(\omega), \end{aligned} \tag{S41}$$

and the chiral component arises from the the second term in the polaritonic spectral density $J_{\rm ch}(\omega)$

$$\begin{aligned} k_{\rm ch} =& \frac{2\pi}{\hbar}\int_0^\infty d\omega\ J_{\rm ch}(\omega)\rho_{\rm vib}(\omega) \\ =& 4\mu_0\int_0^\infty d\omega\ \omega{\rm Im}\big[\mathbf{m}_{\rm CT}\cdot\nabla\times{\rm Im}\overline{\overline{\mathbf{G}}}(\mathbf{r}_{\rm M},\mathbf{r}_{\rm M},\omega)\cdot\mathbf{d}^*_{\rm CT}\big]\rho_{\rm vib}(\omega). \end{aligned} \tag{S42}$$

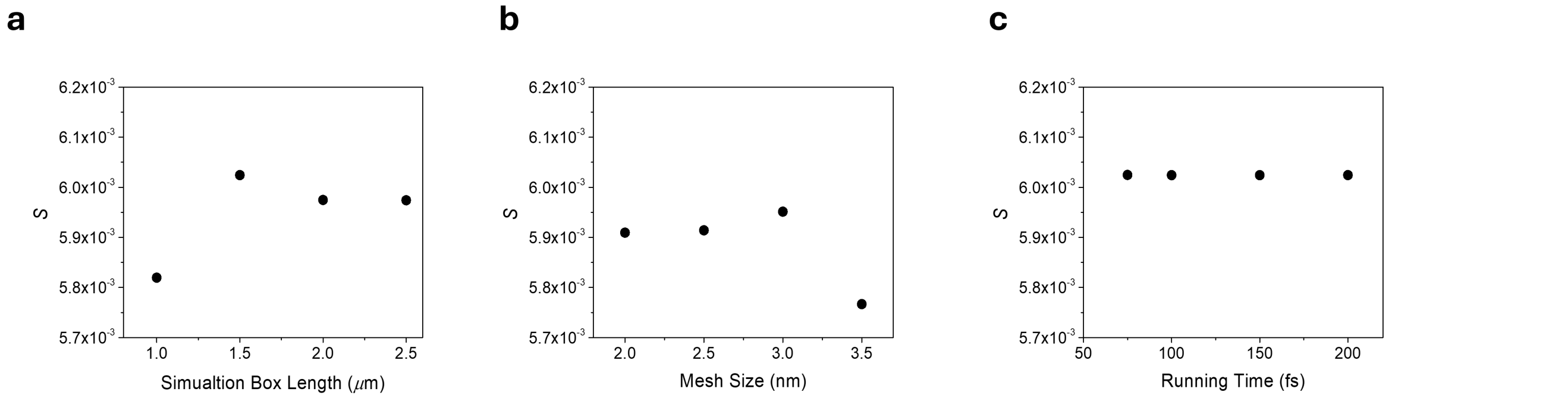

Figure S1: Characterization of numerical accuracy. **a** Dependence of simulation box lengths. **b** Dependence of mesh sizes. **c** Dependence of running times. When varying one parameter, the other parameters remain unchanged from those specified in the Methods section.

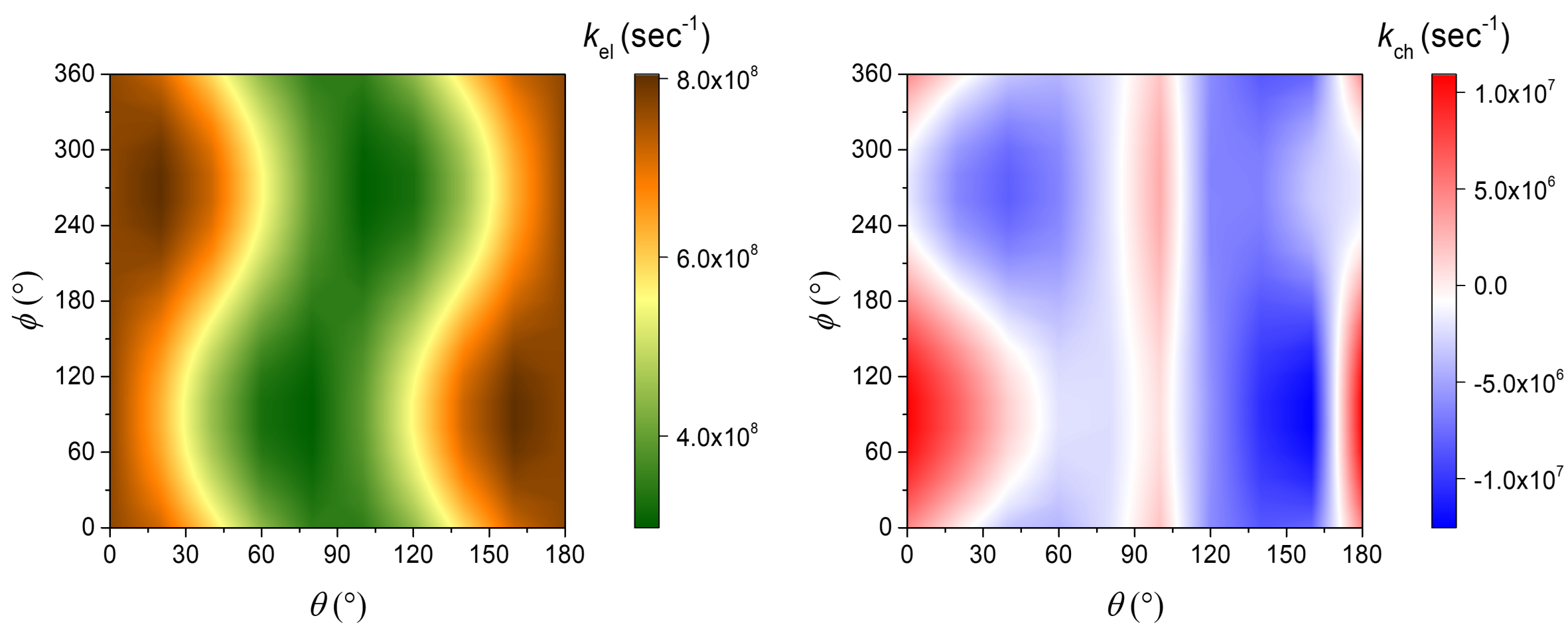

Figure S2: Orientation dependence of k_{el} and k_{ch} for the molecule positioned at the center of the helix.

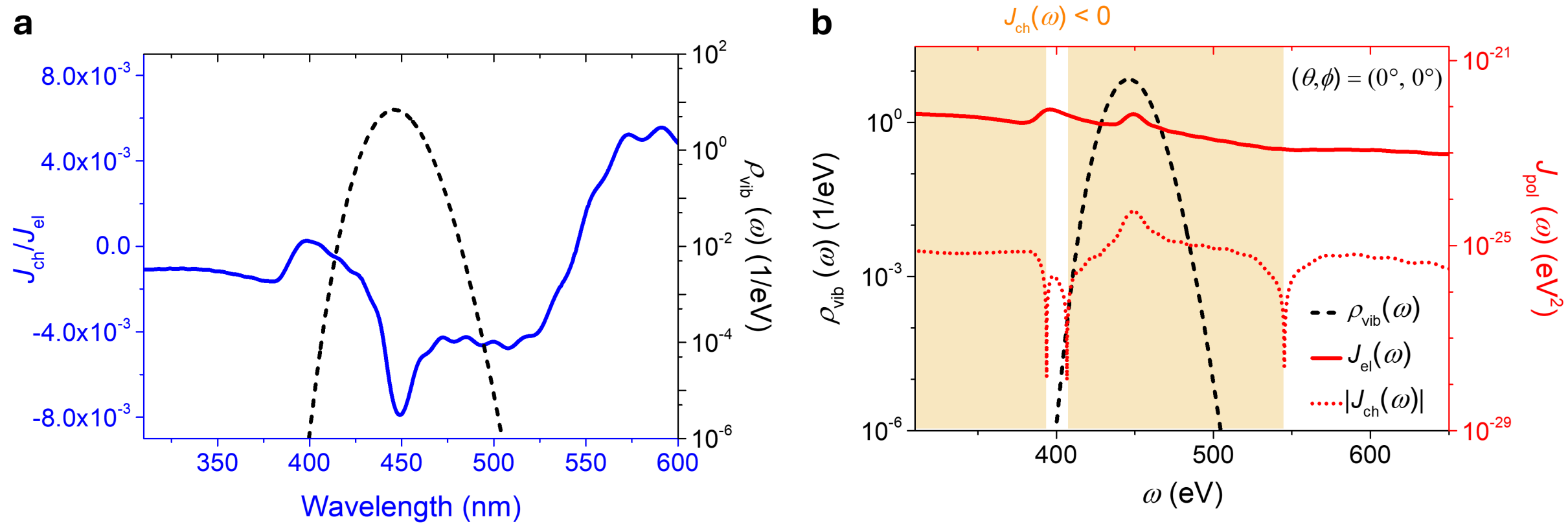

Figure S3: Spectral dependence of **a** J_{ch}/J_{el} and **b** J_{ch} and J_{el} for the molecular electric dipole aligned along the z-axis. The black dashed line is the vibrational transition DOS. The orange regions in **b** are the ranges that $J_{ch} < 0$.

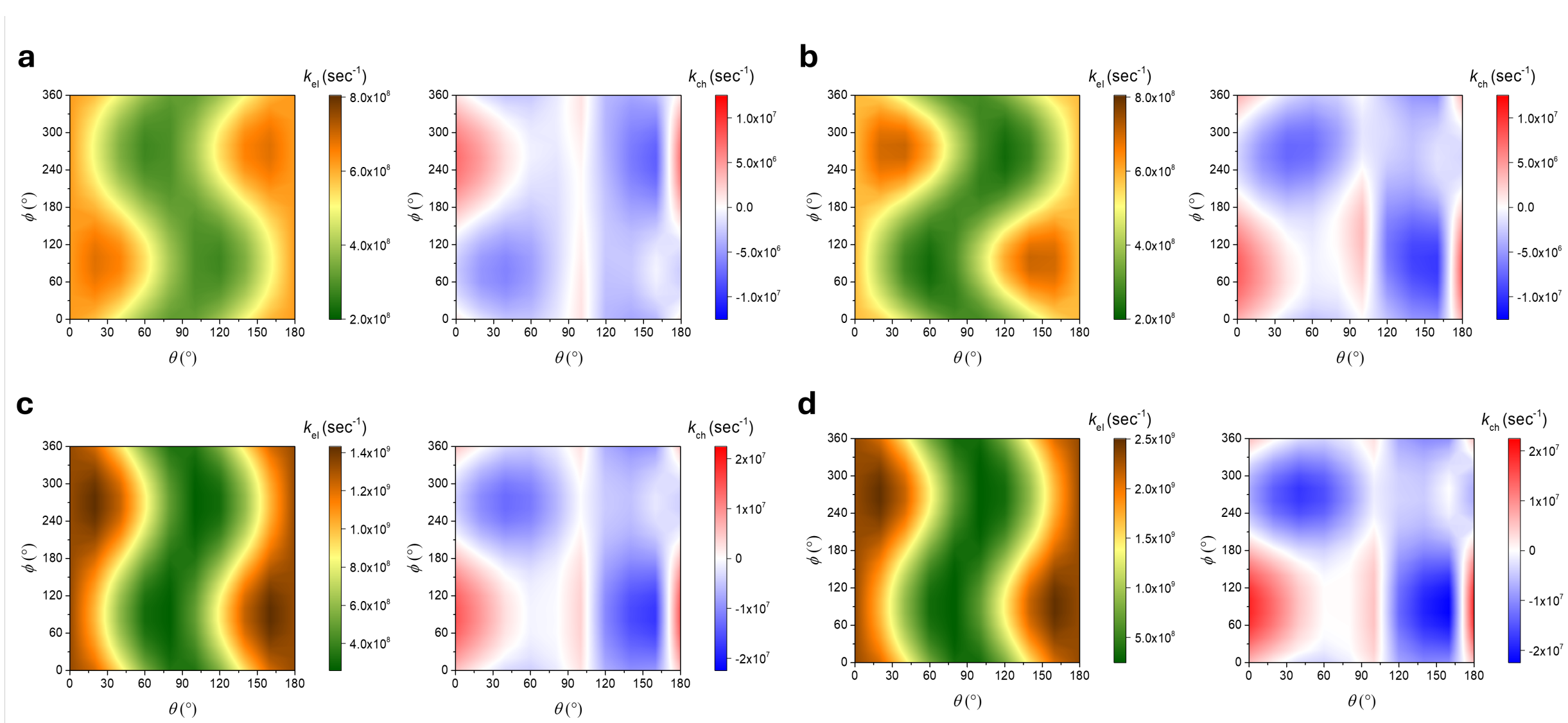

Figure S4: Spatial dependence of the k_{el}-orientation and the k_{ch}-orientation maps for the molecule located at (x,y,z) = **a** (0 nm, 0 nm, 45 nm), **b** (0 nm, 0 nm, 90 nm), **c** (10 nm, 0 nm, 0 nm) and **d** (20 nm, 0 nm, 0 nm). The origin is defined at the center of the helix.

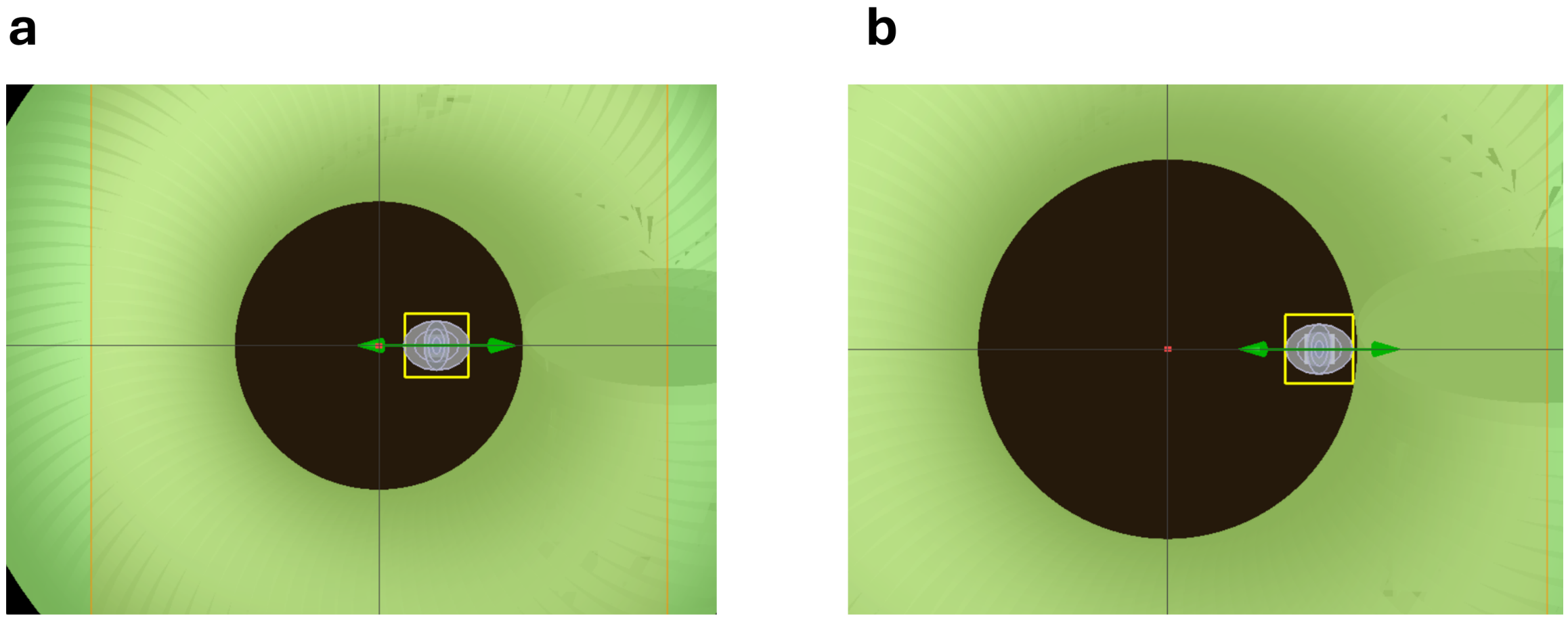

Figure S5: Cross sections of the system in the xy plane ($z = 0$) for the molecule positioned at **a** $(10\ \mathrm{nm}, 0\ \mathrm{nm}, 0\ \mathrm{nm})$ and **b** $(20\ \mathrm{nm}, 0\ \mathrm{nm}, 0\ \mathrm{nm})$. The molecular positions are represented as the center of the arrow. The yellow box indicates the monitored transmission box.

References

(1) Semenov, A.; Nitzan, A. Electron transfer in confined electromagnetic fields. *J. Chem. Phys.* **2019**, *150*, 174122.

(2) Nitzan, A. *Chemical dynamics in condensed phases: relaxation, transfer and reactions in condensed molecular systems*; Oxford university press, 2006.

(3) Gruner, T.; Welsch, D.-G. Green-function approach to the radiation-field quantization for homogeneous and inhomogeneous Kramers-Kronig dielectrics. *Phys. Rev. A* **1996**, *53*, 1818.

(4) Dung, H. T.; Knöll, L.; Welsch, D.-G. Three-dimensional quantization of the electromagnetic field in dispersive and absorbing inhomogeneous dielectrics. *Phys. Rev. A* **1998**, *57*, 3931.

(5) Buhmann, S. Y. *Dispersion Forces I: Macroscopic quantum electrodynamics and ground-state Casimir, Casimir–Polder and van der Waals forces*; Springer, 2013.

(6) Rapp, C.; Franz, J. C.; Buhmann, S. Y.; Franca, O. Purcell effect in chiral environments: A macroscopic QED perspective. *Phys. Rev. A* **2025**, *112*, 013109.

(7) Scheel, S.; Buhmann, S. Y.; others Macroscopic quantum electrodynamics-concepts and applications. *Acta Phys. Slovaca* **2008**, *58*, 675–809.

(8) Ding, W.; Hsu, L.-Y.; Schatz, G. C. Plasmon-coupled resonance energy transfer: A real-time electrodynamics approach. *J. Chem. Phys.* **2017**, *146*.

(9) Portela-Pino, J.; Amorim, I.; Chiussi, S.; Salonen, L. M.; Ozcelik, A.; Aranda, D.; Uusitalo, J.; Peña-Gallego, Á.; Santoro, F.; Kolen'ko, Y. V.; others Parallel alignment of electric and magnetic transition dipole moments in chiral additives can boost the oxygen evolution reaction. *Commun. Chem.* **2026**,

(10) Chang, H.-T.; Zhang, P.-P.; Cheng, Y.-C. Criteria for the Accuracy of Small Polaron Quantum Master Equation in Simulating Excitation Energy Transfer Dynamics. *J. Chem. Phys.* **2013**, *139*, 224112.

(11) Hsu, L.-Y.; Tsai, T.-W.; Jin, B.-Y. Transport through a Mixed-Valence Molecular Transistor in the Sequential-Tunneling Regime: Theoretical Insight from the Two-Site Peierls–Hubbard Model. *J. Chem. Phys.* **2010**, *133*, 144705.

(12) Novotny, L.; Hecht, B. *Principles of nano-optics*; Cambridge university press, 2012.

(13) Wei, Y.-C.; Hsu, L.-Y. Cavity-Free Quantum-Electrodynamic Electron Transfer Reactions. *J. Phys. Chem. Lett.* **2022**, *13*, 9695–9702.

(14) Buhmann, S. Y.; Knöll, L.; Welsch, D.-G.; Dung, H. T. Casimir-Polder forces: A nonperturbative approach. *Phys. Rev. A* **2004**, *70*, 052117.

(15) Wei, Y.-C.; Hsu, L.-Y. Wide-dynamic-range control of quantum-electrodynamic electron transfer reactions in the weak coupling regime. *J. Phys. Chem. Lett.* **2024**, *15*, 7403–7410.
